# Transnasal humidified rapid insufflation ventilatory exchange during bronchoscopy in severe pulmonary hypertension due to Gerbode defect

**DOI:** 10.1002/rcr2.519

**Published:** 2020-03-04

**Authors:** Lucas Upperman, Thomas Gildea, Ursula Galway

**Affiliations:** ^1^ Department of Anesthesiology Cleveland Clinic Foundation Cleveland OH USA; ^2^ Department of Pulmonary Medicine Cleveland Clinic Foundation Cleveland OH USA

**Keywords:** Apnoeic oxygenation, bronchoscopy, dexmedetomidine, high‐flow nasal oxygenation, pulmonary hypertension

## Abstract

Transnasal humidified rapid insufflation ventilatory exchange (THRIVE) has been shown to prevent desaturation and prolong apnoeic time in the perioperative setting. Previously, pulmonary hypertension was not studied using this technique, due to concern for hypercapnia and resultant transient worsening of mean pulmonary artery pressure. We describe a patient with severe pulmonary hypertension (mean pulmonary artery pressure of 82 mmHg) who underwent bronchoscopy using deep sedation with THRIVE. There were no recorded desaturation events and no post‐operative signs of hypercapnia. This case report demonstrates a novel use of THRIVE in a patient with severe pulmonary hypertension.

## Abstract 2

Transnasal humidified rapid insufflation ventilatory exchange (THRIVE) has been shown to prevent desaturation and prolong apnoeic time in the perioperative setting but has not been used in patients with pulmonary hypertension due to concern for hypercapnia and resultant transient worsening of mean pulmonary artery pressure. Here, we describe a patient with severe pulmonary hypertension (mean pulmonary artery pressure of 82 mmHg) who underwent bronchoscopy using deep sedation with THRIVE with no recorded desaturation events and no post‐operative signs of hypercapnia.

## Introduction

Transnasal humidified rapid insufflation ventilatory exchange (THRIVE) delivers warmed and humidified oxygen via nasal cannula at up to 70 L/min. High‐flow oxygen delivery has been demonstrated to prolong the apnoeic window, or the time between induction of anaesthesia until airway securement or emergence from anaesthesia and restoration of spontaneous respirations 1. THRIVE using OptiFlow (Fisher & Paykel Healthcare, Auckland, New Zealand) was shown to prevent desaturation below 90% in apnoeic times up to 65 min in patients presenting for otolaryngology surgery with known or predicted difficult airway 1. THRIVE has been repeatedly shown to prevent desaturation; however, there is concern for accumulation of carbon dioxide (CO_2_) in the absence of ventilation 2, 3.

Pulmonary hypertension poses a high risk in patients undergoing anaesthesia. Hypercapnia and hypoxia can exacerbate pulmonary hypertension. Due to concerns for hypercapnia with THRIVE, pulmonary hypertension has been a common exclusion criterion 2. Acute increases in CO_2_ and resultant decreases in pH have been shown to transiently worsen mean pulmonary artery pressure in patients with pulmonary hypertension 4.

We present a case where THRIVE was used during bronchoscopy in a patient with known severe pulmonary hypertension.

## Case Report

The patient was a 28‐year‐old, 57‐kg, female with severe pulmonary hypertension secondary to Gerbode defect, a left to right shunt between the left ventricle and the right atrium 5. Although she recalled dyspnoea and fatigue as a child compared to her peers, she was not evaluated by a physician until age 18 years. Right heart catheterization 18 months prior to presentation showed suprasystemic pulmonary hypertension, with pulmonary artery pressure of 117/58 mmHg, mean pulmonary artery pressure of 82 mmHg, cardiac output via thermodilution method of 4.53 L/min, cardiac index via thermodilution method of 2.80 L/min, pulmonary vascular resistance of 16 Woods units, and transpulmonary gradient of 74 mmHg. It was decided she should pursue lung transplantation; however, during workup she developed a persistent dry cough. Computed tomography (CT) chest was notable for irregular pulmonary nodules. Repeat CT upon presentation showed progression to cavitary lesions, for which bronchoscopy was planned for investigation. At the time of presentation, medications included sildenafil 80 mg morning and night, and 60 mg afternoon; treprostinil 48 mcg via nebulizer every 4 h; and ambrisentan 10 mg daily. She required 3 L of oxygen at rest and 6 L with exertion. She was unable to lay in the supine position due to shortness of breath.

Bronchoalveolar lavage under moderate sedation provided by the pulmonologist without an anaesthesia team was planned for further evaluation of newly discovered cavitary lesions. In light of the severity of pulmonary hypertension, it was believed diagnostic discovery outweighed procedural risks. It was decided an anaesthesia team would allow safer administration of sedation for the bronchoscopy. Our main anaesthetic concern with administration of sedation was the avoidance of hypercarbia and hypoxaemia which could exacerbate her pulmonary hypertension. We decided the best approach was to topicalize the airway and provide sedation along with high‐flow nasal oxygen which potentially could lessen her chances of developing hypoxia and hypercarbia. We hoped to avoid general anaesthesia which would be poorly tolerated given the extent of her pulmonary hypertension.

On arrival to the bronchoscopy suite, she had a pre‐existing peripherally inserted central catheter and a midline in situ in the contralateral upper extremity. Non‐invasive blood pressure monitoring was used to record blood pressure every 5 min. Three‐lead electrocardiogram (EKG) and peripheral oxygen saturation monitoring were used continuously. The decision was made not to pursue invasive haemodynamic monitoring due to the short predicted length of procedure, and low level of sedation to be given. Airway was topicalized with 4% lidocaine, first via a nebulizer and subsequently with direct application using an atomizer, and finally direct application on vocal cords via bronchoscope. Dexmedetomidine was the sedative of choice to avoid suppression of spontaneous respiration. The patient was placed on OptiFlow intranasal delivery of oxygen at 70 L/min while in the sitting position. End‐tidal CO_2_ (ETCO_2_) was not monitored due to the incompatibility with high‐flow nasal oxygen. To avoid hypotension and bradycardia, a low‐dose bolus of 0.5 μg/kg intravenous dexmedetomidine was given over 15 min. She also received a single dose of 0.5 mg intravenous midazolam for anxiolysis at induction followed later by an additional dose of 1.5 mg midazolam in divided doses. Dexmedetomidine continuous infusion ran at 0.7 mcg/kg/h following initial bolus. Despite adequate topicalization she continued to cough, thus 100 mcg intravenous fentanyl was given in four divided doses. Intravenous ondansetron (4 mg) was given prior to conclusion of the bronchoscopy.

Total time from induction to emergence was 23 min. Spontaneous respirations were preserved throughout, with respiratory rate constant at 18. Oxygen saturation was maintained above 96% for the entire procedure. Mean arterial pressure was maintained at 80–90 mmHg throughout. Heart rate ranged from 80 to 100 beats per minute, with an elevation to 120 upon insertion of bronchoscope. Throughout the procedure, the patient was responsive to verbal stimuli. Patient was transported from bronchoscopy suite to post‐anaesthesia recovery unit, where she met all criteria for discharge to regular nursing floor. There were no complications noted post‐operatively. She was very satisfied with the procedure and had minimal recall.

## Discussion

Pulmonary hypertension poses a high risk for general anaesthesia. These patients have an elevated mean pulmonary artery pressure, and in the face of acute acidosis and elevated CO_2_ there is concern for transient worsening of pulmonary hypertension 4, 6. For this reason, pulmonary hypertension has previously been excluded from trials using THRIVE; however, there is good reason to minimize mechanical ventilation and general anaesthesia where applicable in these patients. Among patients with pulmonary hypertension undergoing non‐cardiac surgery, elevated right atrial pressure, 6‐min walking distance less than 399 m, emergency surgery, and perioperative vasopressor use were all risk factors for major complications including haemodynamic instability, heart failure, post‐operative sepsis, respiratory failure, prolonged post‐operative mechanical ventilation, and prolonged intensive care unit stay 7, 8. In consideration of these factors, general anaesthesia is best avoided in favour of regional or neuraxial anaesthesia where applicable; however; it is important to regard standard contraindications such as patient refusal and risk of bleeding. Through selection of patients without other major comorbidities undergoing procedures and surgeries of short duration, it may be possible for THRIVE to decrease post‐operative complications.

Apnoeic oxygenation occurs when a passageway exists between the distal, gas exchanging portion of the lungs and the exterior environment. THRIVE capitalizes on this by flooding the airway with oxygen 1. Multiple studies have demonstrated the utility of high flow nasal cannula (HFNC) during apnoea to prevent desaturation during short operations 2, 6, 9. Emerging from this data is THRIVE's exceptional utility to prevent desaturation events; however, there remains concern for hypercapnia. In 2017, Gustafsson et al. measured the rate of rise in CO_2_ with and without THRIVE. They found the rate of partial pressure of carbon dioxide in arterial blood (PaCO_2_) rise during apnoea to be 0.43–0.68 kPa/min in the absence of high‐flow oxygen, whereas the rate of rise of ETCO_2_ rise was 0.12 kPa/min and the rate of PaCO_2_ rise was 0.24 kPa/min while using THRIVE 6. These findings showed that high‐flow oxygen decreases the rate of CO_2_ rise in apnoeic patients, and that capnography underestimates accumulation of CO_2_ at an increasing rate over time. It has also been theorized that preservation of spontaneous respiration may play a role in decreasing CO_2_ accumulation in anaesthetised patients 2.

HFNC was successful in preventing desaturation in our patient partly due to the use of dexmedetomidine to preserve spontaneous respirations. Dexmedetomidine loading doses have been shown to cause significant systemic vasoconstriction while sparing pulmonary vasculature and is therefore safe for patients with pulmonary hypertension 10. Dexmedetomidine has been used in pulmonary hypertension under monitored anaesthesia care for the avoidance of hypoxic vasoconstriction 11. Furthermore, HFNC was shown to produce flow‐dependent positive pharyngeal pressures of 2–8 cm H_2_O, reducing the rate of CO_2_ rise via dead space washout 12, 13. HFNC has been proven to be more effective at oxygenation than high‐flow face mask when oxygen is delivered above 40 L/min in bronchoscopy cases 13. In the largest review of HFNC oxygen delivery to date, a complication rate of 5.5% was reported, all of which were transient (<60 sec) and immediately correctable 14.

The major limitation in this case is the lack of CO_2_ monitoring. As discussed above, arterial blood gas sampling is likely to be a more accurate representation of PaCO_2_; however, ETCO_2_ via capnography has been used previously to demonstrate CO_2_ accumulation. Although this patient did not demonstrate effects of hypercarbia following the procedure, CO_2_ measurement would have been a useful quantitative measure. Further studies should employ arterial blood gas sampling in patients receiving high‐flow oxygenation via nasal cannula while spontaneously breathing to test the hypothesis that this will decrease the rate of CO_2_ accumulation.

In this case, THRIVE was used to deliver high‐flow 100% oxygen to a patient with severe pulmonary hypertension undergoing bronchoscopy while spontaneous respiration was maintained, preventing any desaturation events. There are no previous cases reported using THRIVE in a patient with pulmonary hypertension; however, THRIVE was recently used during a dental procedure on a patient with severe cystic fibrosis under deep sedation with similar successful results 15. While the case by You‐ten et al. demonstrates the efficacy of THRIVE for patients with pulmonary compromise under deep sedation, the case presented here furthers this discussion by including a patient with pulmonary hypertension and the use of dexmedetomidine.

### Disclosure Statement

Appropriate written informed consent was obtained for publication of this case report and accompanying images.
